# Functional Aspects of the EGF-Induced MAP Kinase Cascade: A Complex Self-Organizing System Approach

**DOI:** 10.1371/journal.pone.0111612

**Published:** 2014-11-05

**Authors:** Efstratios K. Kosmidis, Vasiliki Moschou, Georgios Ziogas, Ioannis Boukovinas, Maria Albani, Nikolaos A. Laskaris

**Affiliations:** 1 Laboratory of Physiology, Department of Medicine, Aristotle University of Thessaloniki, University Campus, Thessaloniki, Greece; 2 AIIA Laboratory, Department of Informatics, Aristotle University of Thessaloniki, University Campus, Thessaloniki, Greece; 3 Bioclinic Oncology Unit, Thessaloniki, Greece; Keio University, Japan

## Abstract

The EGF-induced MAP kinase cascade is one of the most important and best characterized networks in intracellular signalling. It has a vital role in the development and maturation of living organisms. However, when deregulated, it is involved in the onset of a number of diseases. Based on a computational model describing a “surface” and an “internalized” parallel route, we use systems biology techniques to characterize aspects of the network’s functional organization. We examine the re-organization of protein groups from low to high external stimulation, define functional groups of proteins within the network, determine the parameter best encoding for input intensity and predict the effect of protein removal to the system’s output response. Extensive functional re-organization of proteins is observed in the lower end of stimulus concentrations. As we move to higher concentrations the variability is less pronounced. 6 functional groups have emerged from a consensus clustering approach, reflecting different dynamical aspects of the network. Mutual information investigation revealed that the maximum activation rate of the two output proteins best encodes for stimulus intensity. Removal of each protein of the network resulted in a range of graded effects, from complete silencing to intense activation. Our results provide a new “vista” of the EGF-induced MAP kinase cascade, from the perspective of complex self-organizing systems. Functional grouping of the proteins reveals an organizational scheme contrasting the current understanding of modular topology. The six identified groups may provide the means to experimentally follow the dynamics of this complex network. Also, the vulnerability analysis approach may be used for the development of novel therapeutic targets in the context of personalized medicine.

## Introduction

Cells use intracellular signalling pathways to dynamically respond to external and internal stimuli [1]. The activation of these pathways, usually through a cascade of protein phosphorylations, alters the cell’s transcriptional and/or metabolic activities to accommodate to new environmental needs. The great importance of intracellular signalling in development, normal function and disease has attracted an ever increasing scientific interest in understanding and regulating its role.

One of the most important and best studied networks is the epidermal growth factor receptor (EGFR) signalling pathway. EGFR belongs to a family of receptor tyrosine kinases that includes three other members (erbB2/HER-2, erbB3/HER-3, and erbB4/HER-4) [2]. It is anchored in the cytoplasmic membrane, composed of an extracellular ligand-binding domain, a short hydrophobic transmembrane region, and an intracytoplasmic tyrosine kinase domain (reviewed in refs. [3], [4]).

EGFR becomes activated by ligand-dependent as well as ligand-independent mechanisms and receptor upregulation (frequent in cancer). The epidermal growth factor (EGF) is one of the seven known ligands that bind to the EGFR [2]. EGF binding induces a conformational change of the receptor ectodomain that allows for receptor homodimerization (or heterodimerization with one of the other members of the family), and autophosphorylation of several tyrosine residues within the COOH-terminal tail of the receptor [5], [6]. As a means of signal attenuation, activated EGFR is down-regulated by internalization and degradation [7]. However, it may also recycle back to the plasma membrane and it has been reported that internalized activated EGFR continues to signal in endosomal compartments forming a second, internalized pathway, parallel to the cytoplasmic one [8]. EGFR autophosphorylation elicits downstream activation and signalling by several other proteins that associate with the phosphorylated tyrosines through their own phosphotyrosine-binding domains. These downstream proteins initiate several signal transduction sub-pathways, including the mitogen-activated protein kinase cascade (MAPK) [9].

Numerous experimental studies have provided us with a modular view of the MAPK organization. Within the cascade, two principal routes are activated following EGFR activation, a Shc-dependent and a Shc-independent, leading to the activation of Ras subfamily members [10], [11]. Activated Ras activates the protein kinase activity of Raf kinase [12]. Raf kinase phosphorylates and activates MEK (MEK1 and MEK2) which then phosphorylates and activates a mitogen-activated protein kinase (ERK). Finally, activated ERK activates and regulates several cellular proteins and nuclear transcription factors to promote MAPK function which includes cell proliferation, differentiation, growth, migration, adhesion and survival [13]. EGFR activation and the subsequent MAPK activation have therefore a central role in the organism’s development and maturation processes [14]. This pathway, when deregulated, results in the development of a number of malignancies [4]. Specific antibodies and small molecules have been developed in the past few years to silence the constitutive activation of the MAPK pathway and are being used in the clinical setting [15]. Their success is however constrained by either the existence of constitutively active downstream proteins or the development of drug resistance.

Understanding the structure and function of MAPK is crucial for the development of new targeted therapies and for overcoming resistance mechanisms. This task has proven a challenging puzzle, as the pathway displays a characteristic complexity via the non-linear interactions of the large number of proteins involved and their extensive crosstalk. Biochemistry and molecular biology have provided us with a bulk of information regarding reaction kinetics, protein to protein interactions and mutational effects. Based on these results, early mass action mathematical models have been constructed [16]–[19] and used as a starting point for more elaborate, data driven models of the pathway [20]–[21]. Sensitivity and control theory studies have shown the dependency of output protein concentration on the Shc-dependent pathway [22], highlighted the central role of Raf activity [23] and examined the role of subsystem redundancy [24]. Another theoretical study predicted distinct temporal patterns of autophosphorylation for different EGFR tyrosine residues demonstrating the network’s inherent complexity [25]. Spatio-temporal aspects of the EGFR pathway have been resolved using multi-compartmental modelling incorporating the experimentally confirmed result that the activities of the phosphatases involved in dampening EGFR phosphorylation are comparable across different cellular locations [26]. The constitutive activation of EGFR and the underlying regulatory mechanisms have also been investigated in a model driven by phosphoproteomics data [27]. Finally, in more clinically relevant approaches, strategies for successful drug or miRNA applications have been considered using computational modelling [28]–[30].

In this work, we attempt a functional characterization for the EGF-activated MAPK pathway. We examine the pathway from the perspective of complex self-organizing systems, using the time-dependent protein concentrations estimated from the model developed by Schoeberl et al., 2002 [19]. To this end, we employed a framework for mining information from the signals observed from a dynamical and distributed biological system [31], [32]. We use a clustering algorithm to identify functional subgroups within the network and show their internal re-organization when moving from low to high stimulus concentrations. Next, we identify the parameter best encoding for stimulus intensity with mutual information techniques and use it to estimate the impact of removing each protein from the network. Our results may be used for the successful experimental monitoring of the system’s dynamics and as a strategic approach to novel drug development.

## Results

### Subgroup functional re-organization from low to high EGF concentrations

**Figure 1:**
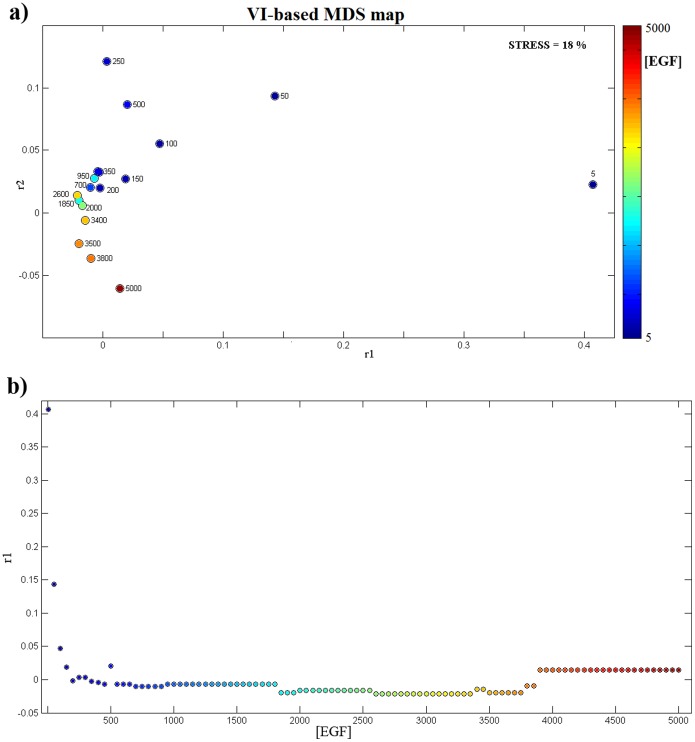
Visual comparison of protein groupings. The groupings for different levels of EGF concentration were compared against each other by means of VI-metric and the obtained results are presented geometrically, by means of MDS, in a space of reduced dimensions. **a)** A 2D display reflecting all the pairwise distances between the 101 distinct groupings. Note that there is overlap between clusterings for consecutive values of EGF concentration. **b)** To resolve this overlap, the 1st dimension of the MDS map (r1), has been plotted as a function of [EGF].

EGFR internalization attenuates the system’s response for high EGF concentrations and amplify it for low EGF concentrations, contributing to the marked output homeostasis of the system displayed for a wide range of stimulus concentrations [19]. Here, we examine the functional re-organization of the proteins when moving from low to high EGF concentrations. We considered that similarity in concentration profiles reflects functional dependence and employed a clustering algorithm to group the proteins. This step was repeated for 101 different levels of constant EGF concentration, ranging from 5 to 5000 molecules/cell, each simulation running for 100 min network time. The derived groupings were compared against each other via an appropriate metric. Figure 1 includes the results from all those comparisons. Each point corresponds to a grouping from a particular EGF level and the proximity between any two points reflects the fact that the proteins were grouped similarly for the corresponding pair of EGF levels. In the upper panel of Fig. 1, a 2D display reflecting all the pairwise distances between the 101 distinct groupings is shown. At the extremities of the graph lie the groupings for EGF concentration of 5, 250 and 5000 molecules/cell. Many concentration levels overlap, signifying identical groupings, and are therefore indistinguishable in Fig. 1, panel a. To resolve this overlap, the 1st dimension of the MDS map (r1), has been plotted as a function of [EGF] in panel b of Fig. 1. A great variability can be observed up to about 500 molecules per cell (also reflected in Fig. 1, panel a). For higher EGF concentrations, variability changes stepwise. For concentrations 3850 molecules per cell and higher, protein groupings remain unchanged.

**Figure 2:**
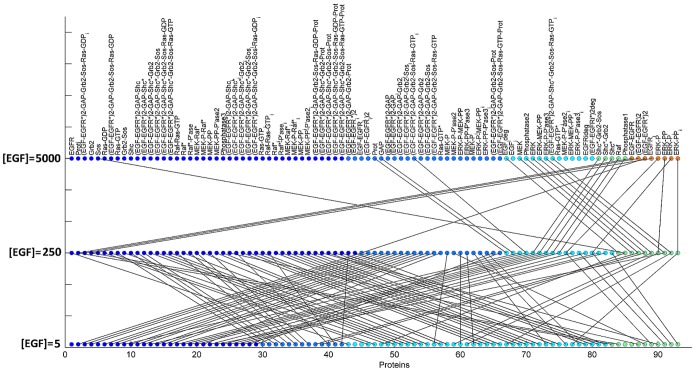
Graphical correspondence between groupings. A schematic representation of how functional groups change from a compositional perspective. The protein groupings for [EGF] = 5, 250, 5000 are compared in pairs. Groups have been ordered in terms of compactness. The color indicates the order of the groups with blue corresponding to the strongest functional cluster. Lines connect the proteins that change group, with the change of [EGF] level.

The extensive re-organization of protein function from a compositional perspective when moving from low to high EGF concentrations is illustrated in Fig. 2. Lines connect the proteins that change groups when different [EGF] levels are compared. Increasing [EGF] from 5 to 250 molecules/cell forces virtually all proteins to change clustering groups. Some group proteins either move together in a new cluster or segregate and re-organize in different groups, marking a new functional role. The changes are less pronounced but still in effect when [EGF] is increased from 250 to 5000 molecules/cell.

**Figure 3:**
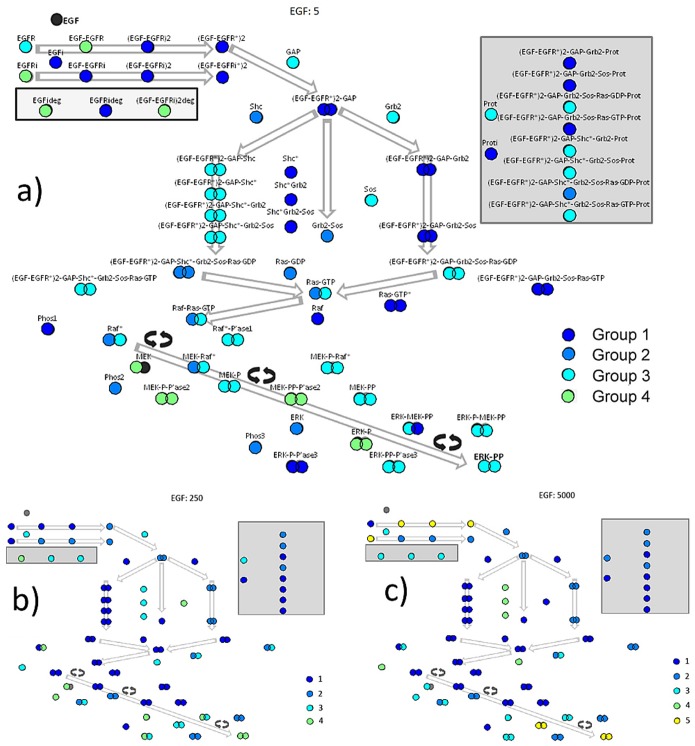
A semantic map of protein-groupings. The functional clusters, derived for three different levels of EGF concentration (a:5, b:250, c:5000), are presented over a graphical outline of the protein network. Proteins in the same group are sharing the same color, while the color of each group indicates the order of the group regarding compactness.

Closer examination of protein groupings formed for the three concentrations of interest, namely 5, 250 and 5000 molecules/cell in panels a, b and c of Fig. 3 respectively reveals functional aspects of the network. We use a schematic representation of the MAPK cascade, reflecting its modular organization. Each circle represents a protein and arrows give the direction of activation or the information flow. Starting from (EGF-EGFR^*^)2-GAP, when circles are shown in doublets side by side, the left denotes the “surface” protein and the right denotes the “internalized”. The Shc-dependent cascade flows (arrows) from left and the Shc-independent flows from the center (through a direct interaction between (EGF-EGFR^*^)2-GAP and Grb2-Sos) and from the right. The flow converges on Ras-GTP and then Raf. Raf, MEK and ERK amplifying loops (circular double arrows) follow and end in the network’s output proteins, ERK-PP and ERK-PP_i_. Protein and protein complex names are identified in panel a, whereas they have been removed in panels b and c for illustration purposes. In Fig. 3, colours indicate the group to which the protein was assigned, with group 1 showing the largest coherence among its members and the last group, the least.

As expected from the results of Fig. 1, very low EGF concentration (Fig. 3, panel a: 5 molecules/cell) has many differences compared to the other two shown. The most prominent is that members of the Shc-independent pathway were assigned in group 1 in panel a, while for higher EGF concentrations members of the Shc-dependent cascade showed the largest coherence. A common observation across all concentrations tested was that groups were formed from proteins belonging to different modules of the network. This analysis revealed a concentration-dependent, cross-modular functional organization. Another common observation was that the output proteins ERK-PP and ERK-PP_i_ always clustered in the same group. This “common-fate” tendency of the output proteins is yet another manifestation of the amazing homeostasis characterizing the MAPK system, probably served by internal functional rearrangement of its elements.

### Consensus clustering recognizes 6 functional subgroups

In an attempt to produce an ‘‘aggregated’’ clustering, that would summarize the common functional organization trends in the overall set of individual clusterings, and hence help in identifying the proteins that share a robust mutual functional-coupling (despite the changes in EGF concentration levels) we adopted a consensus clustering approach [33], [34].

**Figure 4:**
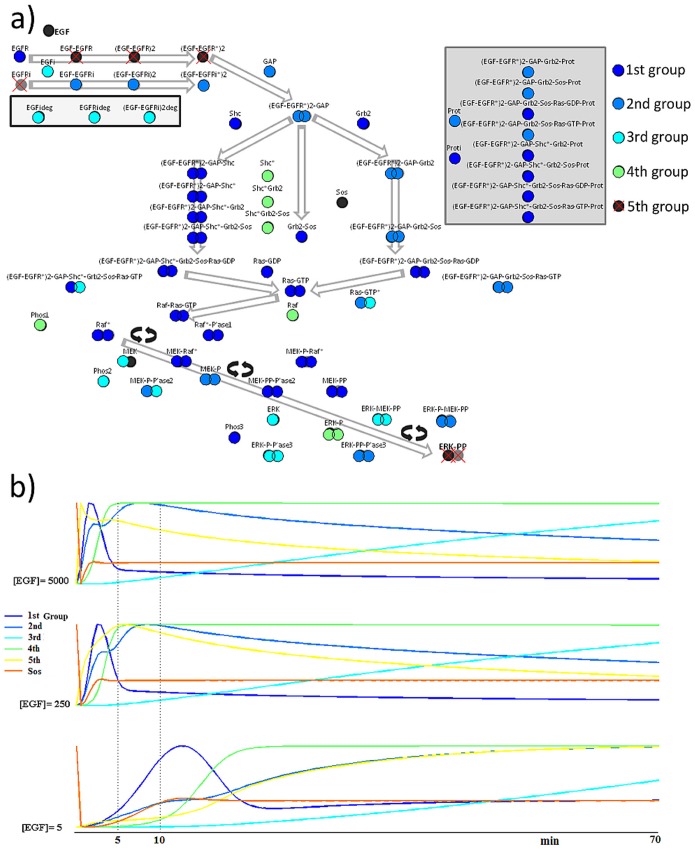
Protein groups from Consensus clustering. a) An “aggregated” clustering in which the protein groups have been ranked according to a score reflecting the mutual coincidence of their members across the 101 different clusterings. The 5th group was the least coherent group and the ‘x’ symbol indicates that its compactness was at the “chance” level. Sos protein appeared as isolated from the rest network. b) The profiles of 6 representative proteins for different levels of EGF concentration.

The results from consensus clustering are shown in Fig. 4 where protein groups have been ranked according to a score reflecting the mutual coincidence of their members across the 101 different clusterings. Five functional groups have been identified with the 5^th^ being the least coherent, below the “chance” level (see Fig. S3). Sos protein appeared as a 6^th^ single member group, isolated from the rest of the network.

In the first group (dark blue in Fig. 4), the concentration of the proteins display a fast rise followed by a relatively fast decline and then a slow degradation towards elimination. Mostly, the key proteins of the Shc-dependent pathway belong in this first group, which also include Ras, Raf and MEK complexes, encompassing both “surface” and “internalized” components. The group is best represented by the concentration time course of the (EGF-EGFR^*^)2-GAP-Shc^*^-Grb2-Sos-Ras-GTP complex, as shown in Table 1.

**Table 1:** Representative proteins.

Representative proteins for each group	
**Group 1**p 1	(EGF-EGFR*)2-GAP-Shc*-Grb2-Sos-Ras-GTP, Raf-Ras-GTP_i, (EGF-EGFR*)2-GAP-Shc*-Grb2-Sos
**Group 2**	(EGF-EGFR*)2-GAP-Grb2-Sos-Prot, (EGF-EGFR*)2-GAP-Grb2-Prot, MEK-P-P'as**e2**
**Group 3**	(EGF-EGFRi*)2deg, EGFRideg, ERK-P-P'ase3
**Group 4**	Shc*, Shc*-Grb2, Shc*-Grb2-Sos
**Group 5**	(EGF-EGFR*)2, EGFR_i, (EGF-EGFR)2
**Group 6**	**Sos**

For each functional group, defined by means of Consensus Clustering, we identified the three more typical proteins. The proteins are listed in order of “typicality”.

The second group (blue in Fig. 4) displays a biphasic response with two peaks, followed by a slow decline. Proteins of the Shc-independent pathway are the main members of this group mainly in internalized EGF-EGFR complexes but also in the MEK family. The (EGF-EGFR^*^)2-GAP-Grb2-Sos-Prot complex resembles the most the average time course of this group (Table 1).

The third group of proteins (light blue in Fig. 4) shows acontinuous slow rise in concentration. The main members are the degraded proteins, Phosphatase 2 (Phos2), MEK, ERK, ERK-P-Phospatase3 and both surface and internalized ERK-MEK-PP. The concentration of the degraded (EGF-EGFR_i_^*^)2 complex is the representative member of this group (Table 1).

In the fourth group (green in Fig. 4), the initial fast rise is followed by near steady state in protein concentration. Activated Shc (Shc^*^) and its complexes with Grb2, Raf, Phosphatase1 (Phos1) and both ERK-P and ERK-P_i_ belong in this group. Shc^*^ undergoes the most characteristic timecourse of this group (Table 1).

The fifth group (black with an “x” in Fig. 4) displays the fastest dynamics, with a biphasic response, with the first spike larger than the subsequent “bump”, and a slow decline later on. Surface EGF-EGFR complexes and the final products of the network, ERK-PP and ERK-PP_i_ belong in this least coherent group. The concentration of the activated dimer (EGF-EGFR^*^)2 is representative of the time course of the proteins in this group (Table 1).

Finally, Sos protein constitutes a single unit group, with an almost instant rise to a maximum value, a sudden drop to near elimination and a recovery to a steady state after a “bump” slightly higher than its steady state final concentration.

### The maximum rate of activated surface or internalized ERK codes for EGF concentration

We sought to determine the parameter of the output of the network, ERK-PP and ERK-PP_i_, best encoding for stimulus concentration. Towards this end, we tested EGF concentrations, from 1 to 500 molecules with increments of 1, from 500 to 1000 with increments of 10 and from 1000 to 5000 with increments of 50 for a total of 629 simulations. We chose to study the effects in finer detail at low EGF concentration based on the results in Fig. 1 where greater variance can be observed in the range of 1 to 1000 molecules. For all simulations, we estimated mutual information between EGF concentration and the following parameters: Area under the curve for ERK-PP (AUC ERK-PP), area under the curve for ERK-PP_i_ (AUC ERK-PP_i_), maximum rate of ERK-PP activation (MaxRate ERK-PP), timing of ERK-PP maximum rate (MaxRate ERK-PP Timing), maximum rate of ERK-PP_i_ activation (MaxRate ERK-PP_i_), timing of ERK-PP_i_ maximum rate (MaxRate ERK-PP_i_ Timing), area under the curve for both ERK-PP and ERK-PP_i_ (AUC ERK-PP+AUC ERK-PP_i_) maximum rate of activation of either ERK-PP or ERK-PP_i_ (MaxRate ERK-PP OR ERK-PP_i_) and minimum timing of either ERK-PP or ERK-PP_i_ (Min Timing ERK-PP OR ERK-PP_i_). The results of this analysis are shown in Table 2. All parameters encoded for stimulus concentration with maximum rate of activation of either ERK-PP or ERK-PP_i_ (MaxRate ERK-PP OR MaxRate ERK-PP_i_) exhibiting the best score. This metric performed marginally better than MaxRate ERK-PP, suggesting that for very low EGF concentrations (from 1–5 molecules/cell) ERK-PP_i_ activates faster than ERK-PP.

**Table 2:** Mutual Information between input-output.

Parameters	Mutual Information Values
AUC ERK-PP	0.714
AUC ERK-PP_i_	0.710
MaxRate ERK-PP	0.831
MaxRate ERK-PP Timing	0.735
MaxRate ERK-PP_i_	0.685
MaxRate ERK-PP_i_ Timing	0.726
AUC ERK-PP+ ERK-PP_i_	0.718
**MaxRate ERK-PP OR ERK-PP_i_**	**0.832**
Min Timing ERK-PP OR ERK-PP_i_	0.731

### Targeting different proteins in the path results in various outcomes

**Figure 5:**
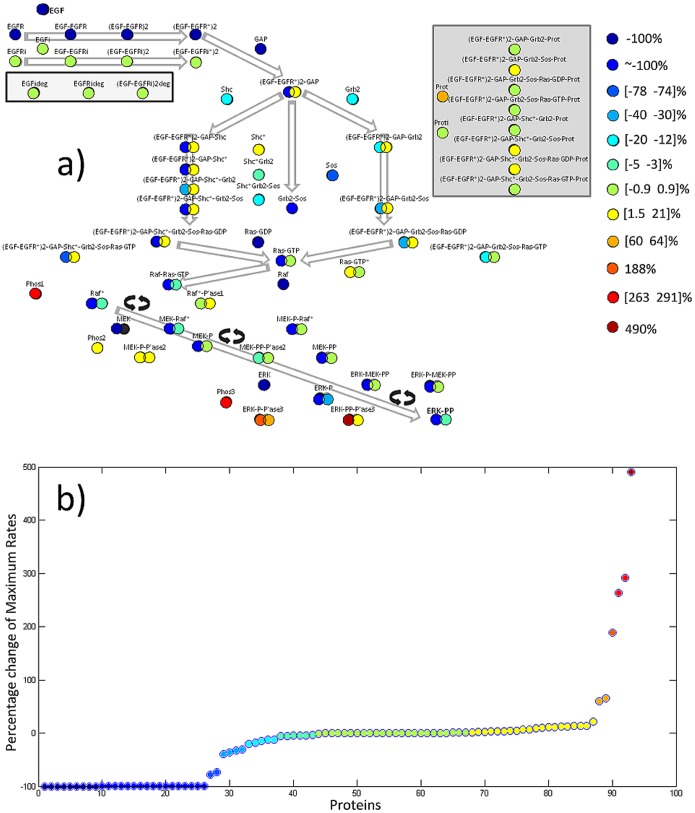
Network vulnerabilty map. Based on simulations, and at the level of [EGF] = 5000, we studied the effect of “removing” each protein on the network functionality. Using the selected (MaxRateERKppOR ERKipp) index, we assigned a score to each protein that reflects the change in that index. These scores were used to rank the proteins and group them according to the type (activation/deactivation) and strength of influences”.

Having determined that the maximum activation rate of either ERK-PP or ERK-PP_i_ is best encoding for stimulus concentration, we studied the effect of removing each protein from the network. For a constant stimulus concentration (EGF = 5000 molecules/cell), we removed one protein at a time and studied the effect on the maximum activation rate of either ERK-PP or ERK-PP_i_. Removal of the protein was achieved by zeroing its initial concentration and its concentration rate equation. Results are shown in Fig. 5. Protein location in the network and color-coded contribution are shown in panel 5a and a rank-based display in panel 5b. The removal of each of a group of nine proteins, namely of the EGFR, EGF-EGFR, (EGF-EGFR)2, (EGF-EGFR*2), GAP, Ras-GDP, Raf, MEK and ERK resulted in complete silencing of the network. On the other end, removal of the ERK-PP-Phosphatase3 resulted in an almost 500% increase in the activation rate of ERK-PP. An increase in activation was observed for virtually all phosphatase members while the removal of most internalized proteins had little effect.

## Discussion

Computer simulations offer a promising, complementary approach for studying complex networks. It has been suggested that applying computational modelling of biochemical signal transduction pathways can help identify drug targets and optimize therapeutics treatments [35]. For instance, a model predicted that an ErbB3 antagonist would inhibit the ErbB-PI3K network activation more efficiently than current marketed therapeutics [36]. This finding led to the development of a novel agent currently tested in clinical trials [37].

The MAP kinase cascade is an important pathway in intracellular signalling with diverse roles in normal function but also in disease manifestation. Pharmacological targeting of a number of its compounds is being used or tested in the clinical setting [29]. Understanding its function will be essential for the successful application of therapeutic strategies, but experimental approaches are limited due to the system’s complexity and extensive crosstalk with other pathways. Based on a data-driven model previously described, we explored the EGF-induced MAP kinase cascade from a complex self-organizing systems perspective, seeking functional aspects of its organization.

We have used a well-developed framework for mining information from the time series of all proteins involved in the model. Proteins displaying similar time courses form functional groups depending on input concentration. Extensive re-arrangement of group members is observed for low EGF concentrations. The dominance of the “internalized” route is restricted to very low, almost negligible, EGF concentrations. The “surface” route determines the network’s response for the vast majority of the cases considered. Re-organization of proteins in a stepwise manner between functional groups was also observed for higher EGF concentration, although less pronounced. Cluster analysis showed a more or less vertical organization with proteins from different modules of the network falling into common groups. Regardless of stimulus concentration, the output proteins of the network always clustered together as a manifestation of its output homeostasis. An integrative view of the cascade’s functional architecture came from our consensus clustering approach. Proteins clustered in 5 groups with characteristic time courses. Sos appears independent from the rest of the network. Grouping the proteins in different functional clusters reduces the complexity of the network. It may also provide the means for experimentalists to follow the dynamics of the network by measuring only the characteristic proteins from each cluster instead of attempting to measure all of them. The formulation of a 6 variable mathematical model able to reproduce the dynamics of the full model would provide solid evidence for our claim and it constitutes the goal of current research. Phosphoproteomic data could verify the time course co-evolution of several proteins under our model’s conditions.

It has been proposed in the past that the activation rate is the important factor in intracellular pathways [19]. Here, we confirm this hypothesis by quantifying the mutual information between stimulus intensity and various other parameters typically measured in experiments. Having established that the activation rate is the key parameter of the network’s encoding ability, we quantified the effect of protein removal. The vulnerability analysis showed that a group of proteins, with members from different functional groups, were absolutely essential for the system’s function. Other proteins have substantial impact in either silencing or enhancing the activation rate of the system’s output. Depending on the desired effect, these proteins may be targets for future drug development. Our analysis also pinpointed a number of proteins with little or no influence on the system’s function.

The interpretation of our results is valid for the model under consideration. However, our methodology is readily applicable to any other model, since it operates on the time series of the constitutive proteins. Also, the results can be extended to arbitrarily complex models displaying the same dynamics for the proteins under consideration as the functional dependences between proteins would remain the same.

The EGFR pathway displays a typical architecture, marked by a range of ligand molecules affecting a number of receptors, a conserved core of interacting proteins and diverse outputs [38]. A detailed description of the network can be found elsewhere [9] and although the resulting map image is fairly complex, it is still a simplification of the biological reality. The model we have used represents only a small fraction of the pathway and that should be kept in mind for the interpretation of our results in the biological context, let aside clinical applications. For example, drugs targeting some of the essential proteins are already in use for therapeutic purposes. Their success is limited by other factors, such as mutational effects of downstream and drug resistive mechanisms. However, modelling should be able to identify essential components and is not intended to be a complete representation of the system under investigation. Simplifying the enormous complexity will be the key for improving our understanding of intracellular pathways. Accounting for mutational effects, resistive mechanisms and virtual drug application is feasible in the context of modelling [39] and will be the aim of our future efforts towards a systems pharmacology approach [40].

## Methods

### Simulations

Source code for the Schoeberl et al. model was downloaded from The CellML project (http://www.cellml.org) and adopted in C. For protein nomenclature and network parameters the reader should refer to the original publication [19]. Compared to the original model, the following rescalings were used: a) concentrations initially in ng/mL were rescaled to molecules per cell, considering a cell volume of 1e^−12 ^L, b) first order rate constants were rescaled from/sec to/minute, c) second order rate constants were rescaled to/(minute x molecule). Simulations typically represented 100 min of network time using an embedded Runge - Kutta – Fehlberg integration algorithm. All algorithms for time-series analysis, clustering, visualization and mutual information were implemented in MATLAB (MathWorks, Natick, Massachusetts, U.S.A.).

### Comparing patterns of activation

The time-dependent concentrations resulting from each simulation are treated as temporal profiles and compared with each other so as to express functional coupling between proteins. In mathematical notation this is accomplished as follows. The time-series c_i_ (t), i = 1,2,…,N, t* = *1,2,…,T (with i running over the proteins in the network and t denoting the discrete time or latency) are first brought to a common scale, by normalizing each one independently so as to range within [0–1]. The normalized activation patterns are depicted as x_i_(t), and collected in a data-matrix **X _[N × T]_** *= *[**x_1_** | **x_2_** |… **x_i_** |… **x_N_**], where ‘|’ denotes a line separator and each row-vector **x_i_** *= *[x_i_ (1), x_i_ (2),…, x_i_ (t),…, x_i_ (T)] ∈ R^T^ corresponds to a protein. Hence **X** represents a point-swarm residing in a multidimensional feature space with axes corresponding to activation-values at particular latencies. The pairwise euclidean distance d_ij_ = ||**x_i_**-**x_j_**||_L2_ quantifies the dissimilarity between the i^th^ and j^th^ proteins. We considered that when two proteins are activated similarly (i.e. their profiles are characterized by a small pairwise distance), they are functionally related. We therefore sought functional groups by means of clustering in the T-dimensional space.

### Clustering via Dominant-sets algorithm

A recently introduced clustering algorithm was employed for detecting cohesive groups of temporal profiles [41]. The algorithm is based on the identification of dominant set of multidimensional points and, when repeatedly executed, facilitates the effective clustering with the additional advantage of adaptively defining the “true” number of clusters. As *dominant set* is characterized the subset of points, in which the overall inter-point similarity is higher than that between the members of the set and the rest of the points.

In a preprocessing step, the inter-profiles distances were transformed to similarities and tabulated in an adjacency matix **A_[N × N]_** with elements A_ij_ = exp (− d_ij_/σ) and A_ii_ = 0. The parameter σ reflects the ‘radius of influence'; in our study was set as equal to the average interpoint distance. The cohesiveness of a given group of points is measured by the overall similarity, which is estimated based on the corresponding entries in **A**. A good cluster-candidate consists of elements that have large values connecting one another in the similarity matrix. Hence, the problem of finding a compact cluster is formulated as the problem of finding a vector **m** that maximizes the following objective function [23]:
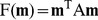
(1)


subject to **m** ∈ Δ, where 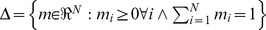.

The algorithmic procedure described in [23], detects the maximally cohesive cluster. Its operation is denoted as: {**m,** F(**m**)} = Dominant_Set(**A**). The vector **m** lists the memberships for all nodes in the set and can be used to identify the exact list of points participating in the dominant-set (by locating the non-zero elements). The second output F(**m**) is the particular value of objective function that measures the cohesiveness of the detected dominant-set. The overall clustering procedure, proceeds in iterative fashion (the matlab code can be found from [42]). The points participating in the dominant-set (at the end of a single execution of the Dominant_Set-routine) are removed from the set and the associated entries in matrix **A** are eliminated. Then, the next dominant-set is delineated by working with the residual adjacency matrix. The detected groups are ranked according to cohesiveness and a diagram summarizing the intermediate steps is produced (for an example, see Fig. S1 and Fig. S2) and exploited for the exact definition of cluster numbers (functional groups). To define a “chance level”, we applied 1000 times the dominant-sets clustering to randomized data (derived by permuting the rows and columns of **A**) and formed the distribution of the cohesiveness values that can appear even in the case of no detectable structure in the point-swarm. The chance level was defined (at significance level α = 0.005) as the threshold value F(m_o_), above which only 0.5% of extracted dominant sets were residing.

### Comparing Functional Groupings via *Variational Information* (VI) measure

To detect possible changes, in terms of network organization, we studied the dependence of functional grouping on the EGF concentation level. We run the computational model 101 times, for different EGF level ranging from 5 to 5000 ng/ml. By applying the dominant-sets algorithm to each one of the resulted point sets { **X^j^** }_j = 1∶101_, we derived 101 distinct clusterings that we systematically compared to each other by means of *VI metric*. Vi is a novel information-theoretic criterion, which has been introduced for comparing two different clusterings of the same data set and measures the amount of information that is lost or gained in changing one clustering to the other [43]. It is a symmetric metric and satisfies the triangular inequality. In our case it was adopted as follows. We denoted the j^th^ clustering output as an N-tuple **c_j_** = [c_j1_,…,c_jN_], c_ji_ ∈Z^+^; e.g. c = [1 3 2 3 2 … 1 1 2 1] indicates that the first and last protein is associated with the strongest functional group, while the second protein is assigned to the group ranked third in terms of cohesiveness. VI was computed for every pair of clusterings:
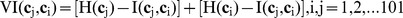
where H(cj) denotes the entropy associated with the particular clustering and I(.,.) denotes the mutual information between two clusterings. The corresponding measurements were tabulated accordingly in a [101×101] distance matrix DVI. The distance-preserving technique of multidimensional scaling (MDS) was then applied to that matrix, i.e. Y[101×2] = MDS(DVI), resulting in a low dimensional scatter-plot in which each point corresponds to a particular clustering and inter-point distances reflect the VI-measurements (see Fig. 1) [44].

### Consensus Clustering

We first form the consensus matrix **P**_[N×N]_ whose entry P_ij_ indicates the number of clusterings in which the i^th^ and j^th^ protein were assigned to the same functional group, divided by number of clusterings (i.e. 101) [33], [34]. Then, by treating **P** matrix as an adjacency matrix **A** ^consensus^, we applied once again the algorithm of dominant-sets (see Fig. S3). The groups detected this way were ranked according to cohesiveness and therefore the most compact group contained the proteins that showed a constant functional dependency (for all range of EGF levels). For each of these functional groups we identified the three most typical (representative) proteins, based on their membership values (the values of vector **m** in eq.1). Finally, to illustrate the relationship between the consensus clustering and the individual ones, we derived an augmented MDS-map in which the ‘projection’ of consensus clustering has been appended (see Fig. S4).

### Mutual Information (MI) estimates for identifying indices of signal propagation

We studied how changes in EGF concentration (considered as ‘‘input’’ signal) influences the concentrations of ERK-PP and ERK-PP_i_ proteins (treated as ‘output’ signal). To this end we simulated the complex signal pathways and, after defining 9 alternative parameters that could be readily deduced from the activation patters of the outputs, quantified the information-transfer based on mutual-information. Using the kth nearest-neighbor mutual information estimation algorithm [45], [46], with k = 5, we measured the strength of association between [EGF] and each of the measured parameters (see table 2). In this way, we identified the observable parameter best-reflecting the involved signal transduction.

### Resilience of protein network

We score each protein based on the percentage-change that is induced to the selected parameter when the particular protein is “deactivated” (by nulling its contribution to the network function).

### “Sensitivity analysis” for the grouping of protein profiles

We performed a sensitivity analysis for the grouping of protein profiles. To test the robustness of the reported self-organization tendency (that is the functional grouping of proteins), with respect to the realization of the particular model, we proceeded as follows. We altered the rate constants of all reactions by adding white Gaussian noise at the beginning of the simulation. The noise was scaled as a percentage of the constant’s original value. Different noise realizations were used for each simulation. For constant input concentration ([EGF] = 5000 molecules/cell), the new protein profiles were fed into the Dominant-sets clustering algorithm and the new groupings were compared against the grouping that corresponded to the unperturbed network. Using the VI-measure as a measure of divergence, so as to measure the dissimilarity between the new and original grouping, we plotted the divergence as a function of noise level. As it can be seen in Figure S5, even for high perturbation (up to 40%), the divergence remains at a reasonable level. The dotted line denotes the divergence from the original grouping of the grouping that resulted from the random permutation of group-labels.

## Supporting Information

Figure S1 **Intermediate algorithmic steps in the execution of dominant-sets clustering algorithm.** a) A sample of protein activation profiles. b) The matrix of all pairwise similarities (between protein activation profiles) in the network. c) The cohesiveness (group-compactness) of the detected groups as a function of rank. Dotted line indicates the chance level (of groups detected in randomized data).
(TIF)

Figure S2 **The grouping of protein activation profiles that corresponds to Fig. S1.**
(TIF)

Figure S3 **Intermediate algorithmic steps in the execution of Consensus Clustering.** a) The [93 × 101] matrix with all the derived clustering lists: each column corresponds to a simulation with different EGF level. b) The consensus matrix. c) The cohesiveness of the detected groups as a function of rank.
(TIF)

Figure S4 **Incorporating the grouping of consensus clustering in the map of** **Fig. 1** **.** The arrow indicates the embedding location of the “aggregated-grouping” in the VI-map.
(TIF)

Figure S5 **“Sensitivity analysis” for the grouping of protein profiles.** Even for high perturbation (up to 40%), the divergence remains at a reasonable level. The dotted line denotes the divergence from the original grouping of the grouping that resulted from the random permutation of group-labels.
(TIF)

File S1 **Network implementation in C and simulation data files.**
(RAR)

File S2 **Matlab source code for data analysis.**
(RAR)
