# Supplementary figures and images for: Functional Aspects of the EGF-Induced MAP Kinase Cascade: A Complex Self-Organizing System Approach
Source: PLoS One. 2014 Nov 5;9(11):e111612. doi: 10.1371/journal.pone.0111612 (PMC4221048; doi:10.1371/journal.pone.0111612)

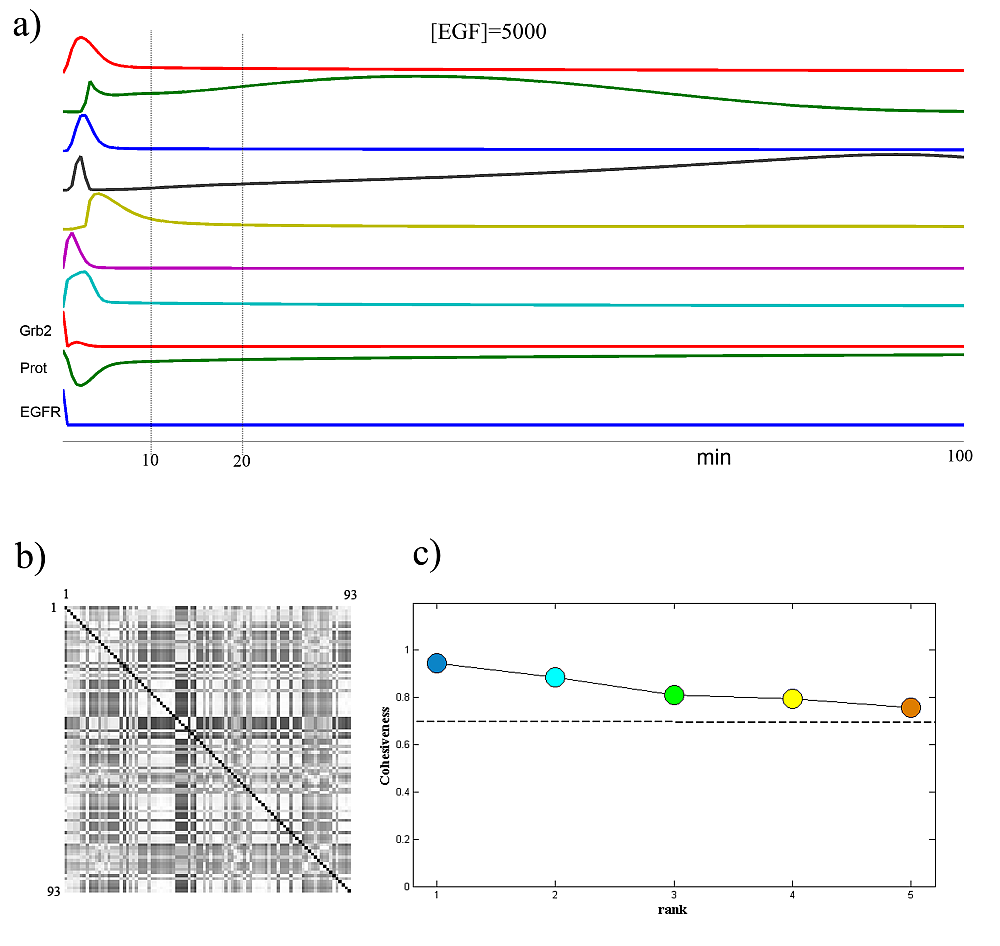

Supplement: Figure S1 — Intermediate algorithmic steps in the execution of dominant-sets clustering algorithm. a) A sample of protein activation profiles. b) The matrix of all pairwise similarities (between protein activation profiles) in the network. c) The cohesiveness (group-compactness) of the detected groups as a function of rank. Dotted line indicates the chance level (of groups detected in randomized data). (TIF) [file pone.0111612.s001.tif]

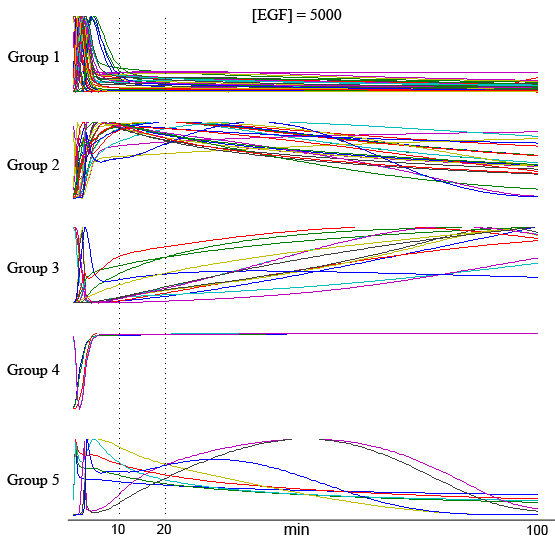

Supplement: Figure S2 — The grouping of protein activation profiles that corresponds to Fig. S1. (TIF) [file pone.0111612.s002.tif]

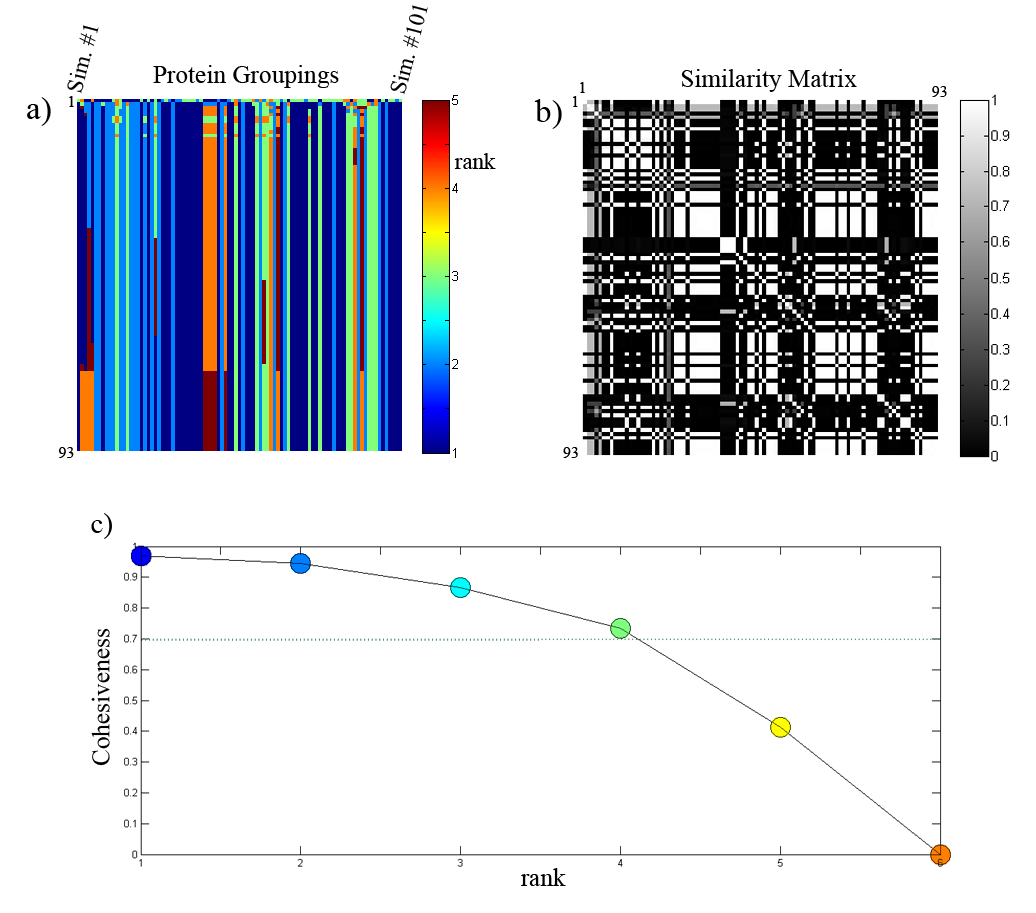

Supplement: Figure S3 — Intermediate algorithmic steps in the execution of Consensus Clustering. a) The [93 × 101] matrix with all the derived clustering lists: each column corresponds to a simulation with different EGF level. b) The consensus matrix. c) The cohesiveness of the detected groups as a function of rank. (TIF) [file pone.0111612.s003.tif]

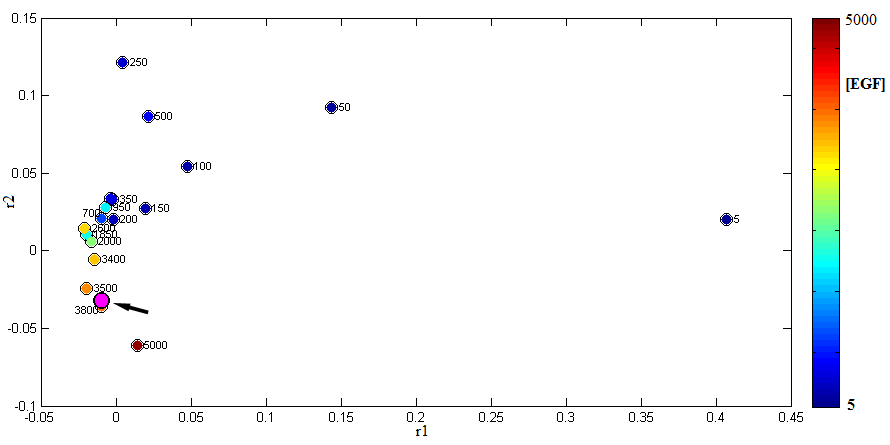

Supplement: Figure S4 — Incorporating the grouping of consensus clustering in the map of Fig. 1 . The arrow indicates the embedding location of the “aggregated-grouping” in the VI-map. (TIF) [file pone.0111612.s004.tif]

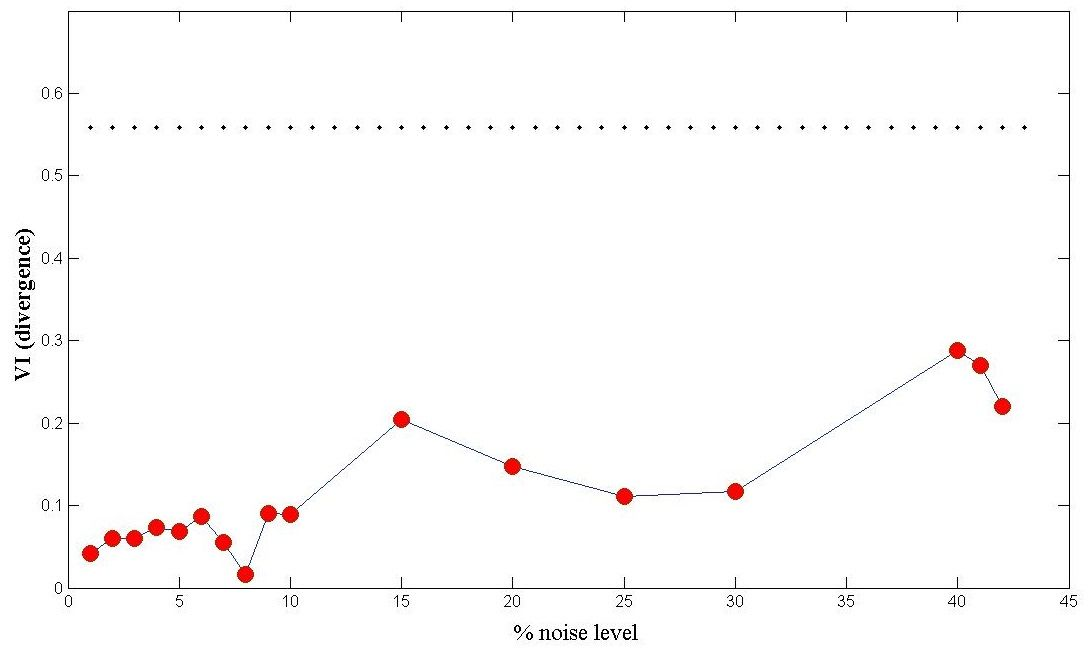

Supplement: Figure S5 — “Sensitivity analysis” for the grouping of protein profiles. Even for high perturbation (up to 40%), the divergence remains at a reasonable level. The dotted line denotes the divergence from the original grouping of the grouping that resulted from the random permutation of group-labels. (TIF) [file pone.0111612.s005.tif]
